# Abstract of the Proceedings of the Buffalo Medical Association

**Published:** 1855-08

**Authors:** Sanford B. Hunt


ART. III.—*Abstract of the Proceedings of the Buffalo Medical Association.*

TUESDAY EVENING, July 3, 1855.

The Association met at the office of Dr. Wilcox.

Present—The President, Dr. Strong, in the chair, and Drs. Wilcox, G. N. Burwell, Hawley, Wyckoff, Baker, Root, Gay, White, Newman, Rochester, Lay, Eastman, Treat, Hubbard, and Hunt.

The minutes of the preceding meeting were read and approved.

*Dr. Burwell* then read a paper on pneumonia as follows:

At the last meeting of the society I reported two cases of pneumonia of the upper lobes of the lungs, both of an intensely inflammatory nature.

I take pleasure in saying that both were able to be about in two or three days after the date of the last note, and have continued to do well in every respect.

I have two other cases of pneumonia to report, both of the upper lobe; and although of sthenic character, not of such a highly inflammatory grade as those reported at our last meeting.

CASE I. Richard O'Shea, laborer, aged 37 years, was taken sick on Tuesday, June 5, with a chill. Continued to work, nevertheless, until the afternoon of June 8, when the chill was repeated with great severity, followed by a high fever, cough, bloody expectoration, and pain in the right side and shoulder. I first saw him at 5, P. M., June 9. He had then the symptoms above detailed; a hot, dry skin and flushed face. Respiration was 36; pulse 112. A loud distinct bronchial respiration was already formed in the upper lobe of the right lung, posteriorly. There was, also, a more than usually abundant crepitant rhonchus. Percussion flat, over the affected lobe. It is worthy of notice that the expectoration, instead of being viscid and scanty, was watery and abundantly mixed with blood. The patient lacks flesh and muscle although a laboring man.

I took from the arm 17 oz. of blood, and directed him to take half a grain of opium, in pill, on every severe paroxysm of coughing. There were symptoms of faintness before I stopped the blood.

June 10, 12, M. The blood drawn yesterday was taken in three parcels: the first, of eight ounces, in a small bowl, had but a trace of the buffy coat; the second, of six ounces, in a tea cup, could just be said to be well buffed; the third, of three ounces, in a tea cup, was both buffed and cupped. The crassamentum of neither parcel had firmness enough to support its own weight.

To-day (nineteen hours from last visit) he had a respiration of 26, easy; pulse 96; some heat of skin, no perspiration; coughs less than heretofore; expectoration quite bloody, but not viscid. The bronchial respiration had lost its clear ringing character, but is quite distinct. The crepitus is still abundant. He has taken since last visit six or eight of the half grain opium pills. To continue them if the cough be troublesome. Dr. Wyckoff saw him with me to-day.

June 11. Dr. Hunt in consultation. The patient's pulse had fallen to 82; respiration 20; no extra heat of skin, some flush of cheeks; expectoration still bloody; rested well last night. Took three or four of the pills.

No further treatment directed.

June 13. Was up and dressed yesterday, fully convalescent.

Case II. Son of William Wilson, aged 4½ years. Taken sick Monday morning, 2 o'clock, June 25, waking from his sleep with a chill. This was followed by a high fever. His principal pain was in his bowels across the epigastric region. The domestic remedies administered by his mother being of no avail, she brought him to my office on Tuesday, June 26. He had then a dry, hot skin, and an active pulse; respiration moderately accelerated, and some cough.

I suspected pneumonia, but on a careful examination I could not locate the disease. I prescribed an active cathartic of calomel and rheubarb, to be followed by an infusion of ipecac in nauseating doses.

June 28, 7, P. M. Was asked to visit him. Although freely physicked and vomited on both of the last two days, there is no abatement of the fever. His nights are very restless, with considerable delirium. He complains much of pain across the stomach, and is vomited by his drinks even. His pulse is 116; respiration 36; cough short and hacking. He has spit up some blood twice, his mother says. His countenance is firm, and for four hours past he has brightened up and appears better than for a day or two. There is a slight dullness on percussion over the upper lobe, right lung, anteriorly, but no decided auscultatory signs to correspond.

Prescribed a solution of nitre and soda.

My better judgment was to bleed him, and I was only deterred from it by the temporary improvement in his symptoms, and because I could not clearly determine, by the physical signs, whether he had pneumonia.

June 29, A. M. The boy had a bad night. Pulse this morning 120; respiration 40; cough short; expectoration scanty, viscid, and *bloody;* skin hot and dry, face flushed, alæ nasi dilated. Pain across stomach still complained of.

The dullness of percussion noticed yesterday, amounts to flatness to-day, and confined to the upper lobe, right lung, anteriorly. There is no corresponding bronchial respiration, nor can any crepitus be developed. There is an increased sharpness and resonance of the respiratory murmur, and which is on a higher key than the natural pitch. The same auscultatory signs are found over the upper lobe, left lung, posteriorly, but without a corresponding dullness on percussion.

Prescribed V. S. ad. 6 oz., to be followed by three grains of Dov. Powd. Repeat the powder every two hours until he is easy.

The bleeding was followed by palor and distinct faintness.

P. M. The boy has had a very comfortable day. Pulse 120, without the fullness and hardness noticed in the morning. Respiration 40; cough much lessened in frequency; no expectoration. The blood drawn this morning is very buffy, and the crassamentum firm and unyielding. The physical signs the same as in the morning, except that on coughing a little crepitus is developed.

To take a Dover's powder if the cough becomes troublesome.

June 30. General condition much as last night. No change in the pulse or respiration; cough lower, no expectoration. Rested well last night. The bronchial respiration in the upper lobe of the right lung is still better developed than heretofore, and a crepitant rhonchus is quite distinct after coughing. Percussion continues flat. The auscultatory signs noticed yesterday morning in the upper lobe, left lung, posteriorly, have disappeared. He has taken two three grain Dover's powders since yesterday, and some of a solution of nitre and soda.

July 1, A. M. Pulse 120; respiration 40; heat of skin still in excess. Percussion unchanged. Bronchial respiration more developed and purer than at any time heretofore. Crepitus heard only after coughing; mucous rhonchus present from the first time.

P. M. There is an exacerbation of fever to-night; pulse still at 120; respiration risen to 52; cough tighter. The mucous rhonchus has disappeared; crepitus is more abundant; bronchial respiration clear.

In consequence of the increase of the fever, and of the rapidity of the breathing, a solution of antimony was given, to be discontinued if it nauseated him too much, or his fever should disappear.

July 2. The boy is very much better. Pulse fallen to 88; the respiration to 28; skin cool. After the second dose of antimony he got nauseated, and broke out into a sweat, and has not had any fever since.

The dullness on percussion remains. The bronchial respiration is quite indistinct, the crepitus heard very slightly only after coughing. No mucous rhonchus.

Discontinue all medicine.

July 3. The boy continues entirely comfortable; has not had any return of fever. Pulse 88; respiration 28. The diseased lung is getting resonant on percussion. The bronchial respiration still exists on a low key; there is no crepitus even after coughing; no mucous rhonchus.

Dismissed convalescent.

Drs. Hunt, Lay, and Wyckoff, did me the favor to see the boy, with me, during the course of his sickness.

On comparing the four cases the following points of interest will be noticed:

1. The fact of pneumonia of such a highly inflammatory character attacking the upper lobes.

2. The upper lobe of the right lung was the seat of the disease in three cases; that of the left lung in one case.

3. In one case the physical signs never showed themselves very distinctly for any length of time; in two not distinctly until the sixth day, and in one case they were all fully developed on the fourth day.

4. The case in which the physical signs were never clearly developed, was the most inflammatory case of all; and did its diagnosis depend on these signs alone, it could as rationally, almost, have been called a pure case of inflammatory fever (old style) as a case of pneumonia.

On the other hand, the case in which the physical signs were fully developed on my first visit, was the least inflammatory of all; the blood taken being the least buffy, and the only one of the four in which the crassamentum would not support its own weight.

5. All four of the cases had bloody expectoration. In three it was viscid. A bloody viscid expectoration in the pneumonia of a child only four and a half years old, will be noticed as something unusual.

In one case the expectoration was watery and not at all sticky or viscid, although abundantly stained or rather mixed with blood. This was in the least inflammatory case, the one in which the blood showed so little buff and tenacity of crassamentum.

6. The boy is the only case which did not have pains resembling pleuritic stitches in the affected side and shoulder.

7. In all the cases venesection was practiced, and for the first time in all, on the fourth day of the disease. In each of the two most inflammatory of the cases, it was practiced three times. In the other two only once in each case. The blood obtained in the first two bleedings in each of the cases repeatedly bled, had a buff of half an inch and over in thickness, and the crassamentum was of such tenacity as to much more than support its own weight when suspended on a stick. That taken in the third bleedings had only a thin covering of buff, although the crassamentum retained its firmness.

In one of the cases of a single bleeding (O'Shea) there was but a light buffy coat to a part of the blood, and a soft crassam[illegible]ntum; in the other (the boy) a decided buff and firm crassamentum.

8. The time between the first bleeding and confirmed convalescence, was as follows: 8 days, 5 days, 4 days, 2½ days. The longest interval was in the most inflammatory case; the shortest in that the least so.

By adding four days to each of these figures, the duration of the disease will be obtained in each patient from the initial chill to convalescence, as follows: 12 days, 9 days, 8 days, 6½ days. The intervals between the last bleeding and convalescence, was 4 days, 4 days, 3 days, 2½ days.

9. In the two cases which were repeatedly bled, the disease did not yield while the blood taken continued to present such a thick buffy coat (half an inch.) In both cases the yielding was instantaneous as it were after the last bleeding. It was also equally sudden in one of the cases bled but once. In the other (the boy) there was no such sudden yielding after bleeding.

10. In two of the cases venesection was wholly relied upon as the curative agent—the only medicine given being a few half grain pills of opium, after bleeding, to allay paroxysms of coughing. In the other two cases free doses of physic were given, and one sickened by the tr. verat. viride, and the other by an infusion of ipecac, all before venesection, but without the least effect in arresting the progress of the disease.

11. In no case was any mercurial given for its constitutional effect; nor were any blisters applied.

In presenting these cases of pneumonia to the society, I wish it to be clearly and distinctly understood, that I do so solely on account of their rarity and interest. It is uncommon (and more so for the last two or three years, than before) to see pneumonia requiring such extensive depletion as practised in two of these cases. To make them examples, therefore, by which all pneumonia indiscriminately should be treated, would lead often to error and disaster.

Even while the two most inflammatory cases were under treatment, I was called to see a woman with pneumonia in the lower lobe of one of the lungs, with flat percussion, well developed bronchial respiration and crepitant rhonchus, whom it would have been bad practice to bleed. And last fall I saw a recent acute case in an active, healthy boy, of some eighteen years of age, which was of such a typhoid character that I treated him with the carbonate of ammonia from the very beginning.

The only safe and prudent way of treating pneumonia, is to let each case depend upon its own indications, and not consider that because a case to day needs a depleting or supporting treatment, or no treatment at all, that the next and all succeeding cases will require the one and same course.

As to the question of the importance of pneumonia, and of its fatality as compared with other dangerous disorders, I would submit that the best way, perhaps, judging of the severity of the disease, is to look over mortuary tables to see how high it ranks among the causes of death. If there be but few which excel or equal it, we may very correctly conclude it to be among the most fatal of diseases.

If, furthermore, we ascertain that this large mortality exists, year after year, without the disease ever prevailing unusually, or as an epidemic, we cannot but look upon it as one of the most important of diseases which can engage our attention.

To apply this test to pneumonia, and for comparison with other diseases, I submit the following figures, few in number, but enough, perhaps, to bear me out in my position:

*First.* In the city of Philadelphia, for five years from 1850 to 1854, inclusive, there have died

| | | | | | | | |
|---|---|---|---|---|---|---|---|
| Of Phthisis Pulmonalis, | 5594 | persons, | or | 120 | in every | 1000 | deaths.* |
| " Convulsions, - - | 2663 | " | " | 57 | " | " | " |
| " Cholera Infantum, - - | 2360 | " | " | 51 | " | " | " |
| " Dysentery, - - | 2175 | " | " | 47 | " | " | " |
| " Pneumonia, - - - | 1923 | " | " | 41 | " | " | " |
| " Scarlatina, - - | 1803 | " | " | 37 | " | " | " |
| " Inf. of the Brain, - - | 1251 | " | " | 24 | " | " | " |
| " Dropsy of the Brain, - | 1247 | " | " | 24 | " | " | " |
| " Croup, - - - | 1064 | " | " | 22 | " | " | " |
| " Typhoid and Typhus Fever, | 1290 | " | " | 27 | " | " | " |
| " Inf. of Stomach and Bowels, | 1040 | " | " | 22 | " | " | " |
| " Bronchitis, - - | 1012 | " | " | 21 | " | " | " |

Total mortality for five years exclusive of still-born, 46,502.

*Second.* In New York city, for thirty weeks preceding the 26th of May last, the total number of deaths was 13,390—still-born included. Of these 1756 were of phthisis, or 131 in every 1000 deaths; and 992 were of pneumonia, or 74 in 1000 deaths.

I regret that I have not on hand for present use and comparison, as full tables of the causes of death in New York city, for a number of years past, as I have of Philadelphia.

*Third.* In our own city, in 1854, a year of pestilence, the order of the most fatal diseases, with the number of deaths respectfully, is as follows:

| | | |
|---|---|---|
| Of Cholera, - - - - - - | 572 | deaths. |
| " Phthisis, - - - - - - - | 266 | " |
| " Cholera Infantum, - - - - - | 187 | " |
| " Convulsions, - - - - - - | 163 | " |
| " Diarrhœa, - - - - - - | 124 | " |
| " Dysentery, - - - - - - | 90 | " |
| " Scarlatina, - - - - - - | 75 | " |
| " Pneumonia, - - - - - - | 73 | " |
| " Dropsy of the Brain, - - - - | 73 | " |

* This statement is made from tables from which still-born cases have been excluded.

| | | |
|---|---|---|
| Of Typhoid Fever, - - - - - - - - | 61 | deaths. |
| " Typhus Fever, - - - - - - - | 34 | " |
| " Measles, - - - - - - - - - | 53 | " |
| " Croup, - - - - - - - - - | 47 | " |

Total number of deaths for the year, 2847, (still-born excluded.)

The number of deaths from phthisis is thus shown to be in the ratio of 93 in 1000 deaths; while that from pneumonia is in the ratio of 25 in 1000 deaths.

If we allow 857 of the total number of deaths as extraordinary and due to the epidemic, the ratio from phthisis will be 133 in every 1000, and of pneumonia 36 in 1000.

For the months of January, February, March, and April, 1855, the account stands thus:

Total deaths, 513, (still-born excluded.)

| | | |
|---|---|---|
| Of Phthisis, - - - - - - - | 84 | deaths. |
| " Convulsions, - - - - - - - | 56 | " |
| " Pneumonia, - - - - - - - | 41 | " |
| " Croup, - - - - - - - - - | 22 | " |
| " Scarlatina, - - - - - - - | 18 | " |
| " Hydrocephalus, - - - - - - | 13 | " |
| " Typhoid Fever, - - - - - - - | 7 | " |
| " Typhus Fever, - - - - - - - - | 12 | " |

Deaths by phthisis, 164 in 1000; by pneumonia, 80 in 1000.

*Dr. Hunt* stated that the results of these statistics were entirely unexpected to him. If pneumonia was really so fatal a disease, the whole subject of its prognosis and treatment required revision. In his own practice it had been, with very rare exceptions, and those confined to the aged, a manageable, and in many cases, even a trivial disease. He had formed his opinions of the disease very much from his own experience. It was possible that this had deceived him, and that a large collection of facts might show a different result. It should be recollected, however, that the statistics of the light treatment pursued by Skoda, Varrentrapp, Dietl, and others, as quoted recently by Dr. Metcalfe, show it to have a mortality of only about five per cent.

*Dr. Burwell* thought that a distinction should be made in cases. In his own practice he saw pneumonia requiring the most active and energetic treatment, while other cases recovered under management far less active.

But it was evident from the figures he had adduced, that it was not a trivial disease, but, on the contrary, ranked very high in the scale of mortality.

*Dr. Rochester* called the attention of Dr. Burwell to the fact that mortuary records were not reliable in the prognosis of disease. In Philadelphia, he knew them to be entirely unreliable except as to the fact of death. The cause of death was certified by almost any one, however incompetent. This was true, also, in this city and in New York, and probably in other places. He did not think that we should form our opinions from them. The recorded experience of physicians was accessible and reliable, and should alone be allowed to decide this question. He had seen but little pneumonia this spring or summer—that which he saw in the winter and early spring in no case required general bloodletting, and but little topical depletion.

*Dr. Burwell* could only speak of what he had seen. The value of general bloodletting in his cases was proved by its success. In cases where the first bleeding failed, a second or third, used later in the disease, was followed by immediate convalescence.

*Dr. Rochester* could not consent to the propriety of venesection after hepatization. He considered it unphilosophical to deplete at a time when the mischief had been consummated, the texture of the lung impaired, and the patient required support to enable him to overcome the disease.

*Dr. Burwell* said that the necessity of venesection after solidification, was made evident by the result. For instance, in the case reported by Dr. Wyckoff, the second and third bleedings were taken very late in the disease, and followed by immediate amendment. And the blood taken at this time was very much buffed and cupped, and the crassamentum remarkably firm.

*Dr. Rochester* replied that the buffy coat was known to be an unsafe element in diagnosis—it occurred in non-inflammatory states, such as chlorosis and tubercle. He did not think that any person should rely upon it.

*Dr. Treat* stated that in the early part of his practice, when living in New England, he was accustomed to ascribe great importance to the buffy coat. He saw much pneumonia there, and was accustomed to bleed very frequently for it; but since he came here he had rarely bled a case, and he had lost faith in the buff. He recollected that Prof. Geo. McClelland said of rheumatism, that the oftener one bled, the more buff he would get—it would go on increasing with repeated venesections.

*Dr. Burwell* considered a thick buffy coat as a very important indication, when accompanied by a firm crassamentum, and by cupping of its surface.

*Dr. Rochester* said that when a student he was taught to bleed, and on commencing practice he did bleed, but he found that cases, even of an acute sthenic type, recovered in his hands now, quite as well as, or better without bleeding, than they did formerly with it.

*Dr. White* moved "That the subject of pneumonia, its pathology, prognosis and treatment, together with the diagnostic value of the buffy coat, and also the papers read upon this subject at this and the preceding meeting, be referred to a committee of three to report at the next meeting."

The motion being seconded, Dr. White remarked that he looked upon impassionate discussion as important. Our judgment, and not our prejudices, should decide for us. He considered isolated cases as of little value. The experience of any one man was insufficient. He commenced practice with the idea that pneumonia was a disease to be combatted by energetic measures, by bloodletting, by calomel, and other active means. His own observation to some extent, but much more the reading of more modern views, had changed his opinions. He had come to regard pneumonia as a trivial disease, requiring the recumbent posture, a careful regimen, and very moderate medication. He was perhaps wrong, and might have to go back to his early notions; at any rate he wished to have the matter discussed and reported on; but his own views, just now, were that bleeding was unnecessary, and that, even if proper at any time, it must be very early in the disease. He concurred with Dr. Rochester as to the value of mortuary reports. It was a matter of considerable moment that we should harmonize and settle on some doctrines, for we were surrounded by men who would use our discrepancies to profit by them. If, after consideration, the society should adopt the sanguinary view of the matter, and go back to the ideas of Cullen, and Rush, and Eberle, he should be under the necessity of differing from them.

*Dr. Burwell* said that he reported these cases on their own merits and success, and not as an advocate of an indiscriminate sanguinary treatment. He treated some cases without venesection. He had seen cases where bleeding would have been fatal. He censured the blind following of routines where one must bleed, or not, in accordance with his favorite author, without regard to the exigencies of the case. He deprecated as much one as the other. If he bled a case to-day, he might stimulate another to-morrow, and mentioned a case of pleurisy where only the recumbent posture was needed. He thought that if Drs. White or Rochester had seen the cases reported, they also would have bled them.

*Dr. White* remarked that he did not criticize these cases or their treatment. He supposed that Dr. Burwell had introduced them as offsets to those of Dr. Metcalfe. He understood him to bring them up as evidence of the necessity of bloodletting and active treatment.

*Dr. Burwell* corrected. He had introduced the *statistics* because his attention was drawn to the subject by Dr. Metcalfe's article. The cases reported were unusual, such as he might not meet again for years.

*Dr. Rochester* again attacked the value of statistics drawn from mortuary reports. Dr. Burwell had supposed that Dr. White or himself would have bled cases similar to Dr. B.'s. For himself he must enter a disclaimer. He could not agree to venesection after hepatization.

*Dr. Burwell* liked the idea of referring the subject to a committee, but did not approve of reporting in one month. Sufficient time should be given to collect facts, no past experience should be introduced, and no mere opinions stated. The committee should give their own cases, and visit patients together. It would require a year to do this, and he hoped that that time would be adopted. As to bleeding after hepatization, he had entire faith in its efficacy and value, is cases with an active, unsubdued pulse and circulation. The case bled in the second stage recovered much more rapidly than the others.

*Dr. Treat* said that bleeding was admissible after solidification, to arouse the absorbents, and hasten the removal of the deposited lymph.

*Dr. White* could not consent to postpone action on his motion for a year. It was not necessary to begin *de novo*. The subject of pneumonia had been studied by others, all the facts were in existence. The committee would be impeded by the unnecessary labor of visiting cases together, and he wanted an early report. We had men enough in the society who were competent to report to-morrow, who had made this subject a specialty, and could benefit us at once.

*Dr. Burwell* did not wish to hamper the committee, but insisted on the propriety and necessity of seeing cases in company.

*The Chair* inquired if this suggestion was offered as an amendment to Dr. White's motion.

*Dr. White* said he could not accept such an amendment, but must leave it to the vote of the society.

The proposition to amend was withdrawn by Dr. Burwell.

*Dr. Wyckoff* thought that due allowance should be made for variation in type. This year it was sthenic, and very inflammatory. Since his case reported, he had seen several other cases of a very inflammatory type.

*Dr. Rochester* again stated that his own number of cases had been limited of late, but he was informed of thirteen cases occurring in the practice of a friend, within the last two months, none of which were bled, but all recovered under a very mild and supporting treatment. Two of these cases were of the upper lobe of the left lung.

The question recurring on Dr. White's motion, it was carried *nem con.* The chair appointed Drs. White, Rochester, and Burwell, as such committee. Dr. White withdrew, and suggested the name of Dr. Flint as his substitute. Dr. Rochester wished the committee to be made up of those who had had no share in the discussion, and also declined. The chair then announced Drs. Flint, Burwell, and Gay, as such committee.

Dr. Burwell wished to decline on the same ground assigned by Dr. Rochester, but finally yielded to the desire of members that he should continue.

*Dr. White* reported a case of placenta prævia:

He was called in consultation, between 10 and 11, A. M., of the 27th of May, to a woman in labor. She was 36 years of age, in her eighth pregnancy; was anæmic, and supposed to have tuberculosis. She menstruated last in December, and was consequently in the eighth month of her pregnancy. The attending physician had been called at 4, A. M., at which time he found the os undilated, with the placenta presenting. There was considerable hæmorrhage, and the attendant introduced the tampon. In the meantime the pains had been light. At 11, A. M., Dr. White found no pulsation of the fœtal heart. The tampon was left *in situ*, and he left to return again at 12½, P. M. At this time no change had occurred in the pains, and he again left. He returned at 2½, P. M., and found that the pains had recurred with considerable flooding. He then proceeded to turn and deliver by the feet, under chloroform. Ergot was given a few minutes before the completion of the labor, to secure contraction. The delivery was accomplished at 3, P. M. The attending physician sat down to maintain pressure by the hand on the uterus, after delivery. Dr. W. examined the organ, and found it well contracted and hard fifteen minutes after delivery. There was then no flowing, and he left her at 3½ in the care of the attendant.

At 5, P. M., he was again called. The attendant informed him that hæmorrhage came on some time previously, that the uterus relaxed under his hand, but did not fill up. He had maintained constant pressure up to this time, and had then resorted to the usual means of controlling uterine hæmorrhage, the cold douche, firm pressure, etc. When Dr. White saw her she had lost much blood, and she died at 6, P. M.

Dr. White remarked that the case had some points of interest. The child was dead when he first saw the case. It was, therefore, a favorable opportunity for adopting Simpson's plan in placenta prævia—to detach the placenta from the uterine wall, and deliver it first. He had only the interest of the mother to consult in this instance, but he had nevertheless preferred the usual operation of turning and removing the child first. He assigned as his reason for so doing, his belief that the hæmorrhage came from the uterine walls, and not from the torn placenta, as taught by Dr. Simpson. He did not think that hæmorrhage from the placenta could be so rapid as it was in these cases, and that, therefore, it must come from the uterine walls. To remove the placenta, then, would leave the source of the evil untouched, or rather increased.

Some discussion then followed as to whether the effect of ergot would not be destroyed by giving chloroform at the same time.

*Dr. White* thought not—it was his habit to do so.

*Dr. Burwell* concurred, and mentioned a case of a lady who in two previous confinements had had dangerous flooding after delivery. In her third confinement she was delivered under chloroform, and ergot was given a quarter of an hour before the birth of the child. In this instance she had no flooding. He thought, however, that the contraction of the uterus for the first fifteen minutes after labor, was no safeguard. The practitioner should remain an hour by his patient.

*Dr. White* agreed, and thought there were few cases where it was safe to leave within an hour.

After some desultory discussion, in which Dr. Treat related a case of placenta prævia attended by great difficulty in dilating the os sufficiently for delivery,

*Dr. Newman* offered the following:

"*Resolved*, That a committee of three be appointed, for the purpose of taking into consideration the propriety of renting and fitting up a suitable room for the meetings of this Association, and to ascertain if a sufficient sum can be raised among the members for the proper furnishing of the same; and also to ascertain the amount of yearly subscription that can be obtained for its subsequent support."

The resolution was adopted unanimously, and the chair appointed Drs. Newman, Wyckoff, and Wilcox, as the committee.

The association then adjourned to meet at the office of Dr. Baker, on the first Tuesday in August.

SANFORD B. HUNT, M. D., Secretary.

